# Surgery and postoperative radiotherapy affect the prognosis of esophageal cancer: A SEER analysis

**DOI:** 10.1097/MD.0000000000032925

**Published:** 2023-03-03

**Authors:** Wenwen Yang, Yanjiang Yang, Xiang Ma, Minjie Ma, Biao Han

**Affiliations:** a The First Clinical College of Lanzhou University, Lanzhou, Gansu Province, China; b Qilu hospital of Shandong University, Shandong University, Jinan, Shandong Province, China; c Department of Thoracic Surgery, the First Hospital of Lanzhou University, Lanzhou, Gansu Province, China; d Gansu Province International Cooperation Base for Research and Application of Key Technology of Thoracic Surgery, The First Hospital of Lanzhou University, Lanzhou, Gansu Province, China.

**Keywords:** esophageal cancer, prognosis, radiotherapy, SEER program, surgery

## Abstract

The principal treatment modalities for esophageal cancer are radiation, chemotherapy and surgery or a combination of them. In some sense, technological advances have tremendously heightened patients’ survival rates. Nevertheless, the debate on the prognostic value of postoperative radiotherapy (PORT) has never ceased. On that account, this study made an effort to probe deep into the effects of PORT and surgery on the prognosis of stage III esophageal cancer. Our study included patients diagnosed with stage III esophageal cancer between 2004 and 2015 through the Surveillance, Epidemiology, and End Results (SEER) program. We performed propensity score matching (PSM) on the basis of whether surgery was carried out and whether PORT conducted. We identified the independent risk factors by multivariate Cox regression and constructed a nomogram model. In this research, we included 3940 patients, and the median follow-up is 14 months: 1932 cases without surgery; 2008 cases with surgery, and 322 cases of them underwent PORT. In the postPSM patient cohort, patients who underwent surgery had a median overall survival rate (OS) of 19.0 (95% confidence interval [CI] 17.2–20.8) and a median cancer-specific survival rate (CSS) of 23.0 (95% CI 20.6–25.3) months, which were remarkably higher than those without surgery (*P* < .001). The OS（*P* < .05）and CSS（*P* < .05）of the patients who underwent PORT were lower than those who did not. Similar results were obtained in the groups of N0 and N1. This study revealed surgery can heighten patients’ survival rate, while PORT could not elevate patients’ survival rate in stage III esophageal cancer patients.

## 1. Introduction

As the 10th most common cancer worldwide, 604,100 (3.1%) new esophageal cancer cases and 544,076 (5.5%) esophageal cancer deaths were reported in 2020.^[1]^ The first choice method of treatment for patients with resectable stage III esophageal cancer is surgery. Nevertheless, survival rate for patients who underwent surgery alone was far from satisfactory.^[2–4]^{Smyth, 2017 #3} Symptoms of stenosis may not appear until the tumor reaches a relatively advanced or even locally metastatic stage.^[3,5]^ Endoscopic resection is feasible in the early stage, chemotherapy can be considered in the advanced stage, and chemotherapy, radiochemotherapy, surgery and combined therapy are recommended in the middle. Surgery alone is less effective and prone to recurrence, therefore needs to be combined with a variety of other adjuvant treatments.^[6–8]^ Postoperative radiotherapy (PORT) is one of the extensively used methods. PORT has been employed in the treatment of esophageal cancer since 1969.^[9]^ Nonetheless, some studies have reported conflicting results regarding the role of PORT.^10–12^ Just as evidently indicated by the results of a randomized controlled trial, PORT elevated patients’ survival rate in stage III esophageal cancer compared with a control group (*P* = .0027).^[10]^ In contrast, in another randomized controlled trial, esophageal cancer patients who underwent PORT had markedly shorter survival rate times than nonPORT patients (*P* = .02).^[11]^ A meta-analysis on 3 randomized controlled trials and 7 retrospective studies persuasively illustrates that PORT can ameliorate overall survival rate (OS) (*P* = .0004) and disease-free survival rate (*P* = .004) in esophageal cancer compared with surgery alone.^[12]^ These studies demonstrates that the role of PORT in esophageal cancer still remains controversial. As a consequence, it remains of interest to evaluate the prognostic value of surgery and PORT. By comparing the role of surgery and PORT, providing clear evidence for the clinical decision-making of clinicians was our primary aim in conducting this study.

## 2. Methods

### 2.1. Study population

**Figure 1.**
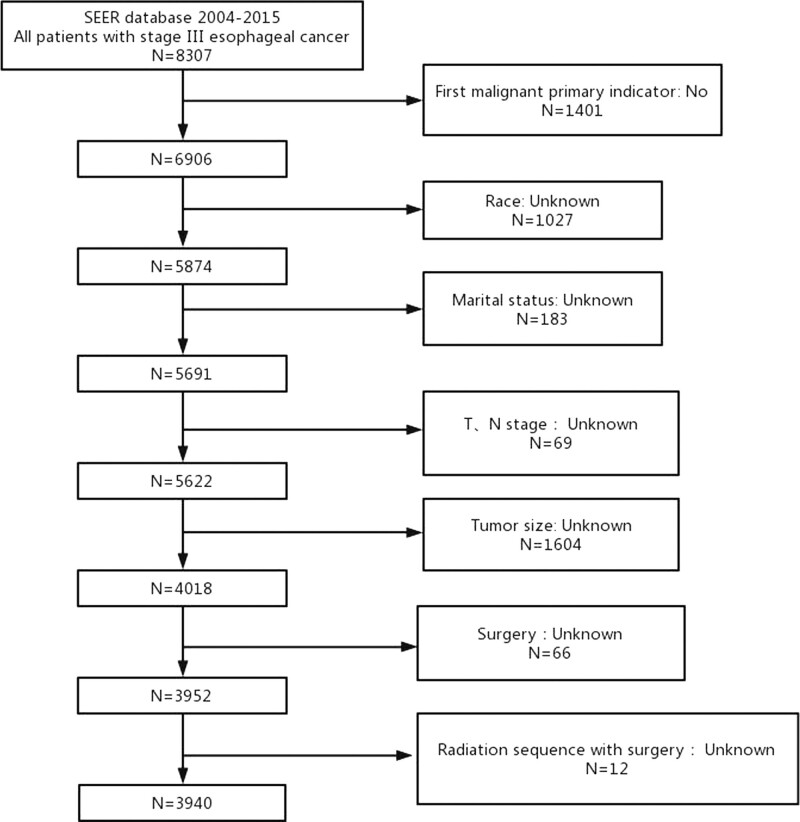
Patient screening flowchart. This figure contains how we screened 3940 stage III esophageal cancer patients from the SEER database. SEER = the Surveillance, Epidemiology, and End Results.

Surveillance, Epidemiology, and End Results (SEER) 18 Regs custom Data (1975–2016) is the data source for this retrospective study. We identified patients diagnosed stage III esophageal cancer from 2004 to 2015 (Fig. 1). Patients included in this exploration had to meet all of the criteria over 18 years of age, tumor size <600 mm, diagnosed with stage III esophageal cancer. The exclusion criteria were as follows not first malignant primary indicator, complete data could not be obtained, patients diagnosed by autopsy. We extracted the following data from the SEER database: age, primary site of tumor, histologic, race, gender, chemotherapy history, T stage, N stage, surgery history, radiation history, tumor size, marital status, radiation sequence with surgery, and follow-up information. We employed OS and cancer-specific survival rate (CSS) as survival rate times analyzed in this investigation. OS is the survival rate time from the day of diagnosis to the day of death from any cause or last follow-up. CSS is an OS measure excluding other causes of death. In this research, we adopted the AJCC 6th edition TNM staging. We use data from public databases and was exempt from institutional review board approval.

### 2.2. Nomogram construction

Univariate and multivariate Cox proportional hazards regression analyses were performed on the postpropensity score matching (postPSM) cohort. On the basis of the above results, we constructed a nomogram using R version 4.1.3.

### 2.3. Statistical analysis and the optimal cutoff value

We compared categorical variables using chi-square or Fisher’s exact test. 1:1 PSM of surgery and PORT were performed separately to eliminate possible effects of other variables. The log-rank test was adopted to evaluate Kaplan–Meier survival rate curves and reported hazard ratios (HR) with 95% confidence interval (CI). In accordance with the results of univariate analysis, we included factors with *P* < .05 into multivariate analysis. SPSS v26.0 (SPSS Inc) and GraphPad Prism v8.0.2 (GraphPad Software, Inc.) were used for Statistical analysis. The optimal cutoffs for tumor size and age were determined in line with X-tile v3.6.1 (Yale University). *P* < .05 was considered statistically significant.

## 3. Results

### 3.1. Baseline characteristics

In this exploration, 3940 stage III esophageal cancer patients were screened from SEER database, of whom 51.0% (n = 2008) underwent surgery and 49.0% (n = 1932) did not. Table 1 summarized the clinical characteristics before and after PSM in line with whether the surgery is performed was conducted. Most surgical and nonsurgical patients were between the ages of 23 and 66 (64.9%; 48.8%) and were both male (84.6%;76.3%), and were both married (68.5%;55.4%); and were both the white (89.9%;77.9%); and were both Poorly differentiated (Grade III) (55.1%;48.4%); and were both T3 stage (81.6%;61.2%); and were both N1 stage (95.7%;83.4%); and all received radiotherapy (77.1%;80.5%) and chemotherapy (83.8%;78.9%). We then compared the clinical characteristics of patients before and after PSM in accordance with whether the PORT is carried out (Table 2). Although most variables did not exhibit statistical differences between PORT(+) and PORT(−) (*P* > .05), we still performed PSM to remove potential effects of other variables. Table 3 displays the clinical characteristics of all stage III esophageal cancer in different N stages.

**Table 1:** Characteristics of patients before and after PSM according to whether or not surgery.

Characteristics	Entire patients					Propensity-matched patients				
	Surgery (+)		Surgery (−)		*P* value	Surgery (+)		Surgery (−)		*P* value
	(n = 2008)	%	(n = 1932)	%		(n = 1192)	%	(n = 1192)	%	
Age at diagnosis					<.001					.343
23–66	1304	64.9	944	48.8		660	55.37	661	55.45	
67–74	473	23.5	481	24.8		329	27.60	305	25.59	
75–97	231	11.5	507	26.2		203	17.03	226	18.96	
Gender					<.001					.756
Female	308	15.3	456	23.6		234	19.63	227	19.04	
Male	1700	84.6	1476	76.3		958	80.37	965	80.96	
Tumor size (mm)					<.001					.799
1–44	926	46.1	647	33.4		462	38.76	472	39.60	
45–70	764	38.0	807	41.7		484	40.60	468	39.26	
71–550	318	15.8	478	24.7		246	20.64	252	21.14	
Marital status					<.001					.313
Unmarried	632	31.4	861	44.5		475	39.85	450	37.75	
Married	1376	68.5	1071	55.4		717	60.15	742	62.25	
Race					<.001					.573
White	1806	89.9	1506	77.9		1021	85.65	1005	84.31	
Black	114	5.6	283	14.6		95	7.97	109	9.14	
Other	88	4.3	143	7.4		76	6.38	78	6.54	
Grade					<.001					.857
Well differentiated; Grade I	82	4	116	6.0		66	5.54	64	5.37	
Moderately differentiated; Grade II	779	38.7	843	43.6		493	41.36	485	40.69	
Poorly differentiated; Grade III	1108	55.1	936	48.4		605	50.76	620	52.01	
Undifferentiated; anaplastic; Grade IV	39	1.9	37	1.9		28	2.35	23	1.93	
T-stage					<.001					.372
T3	1730	81.6	1184	61.2		923	77.43	942	79.03	
T4	278	13.8	748	38.7		269	22.57	250	20.97	
N-stage					<.001					.464
N0	86	4.2	320	16.5		86	7.21	76	6.38	
N1	1922	95.7	1612	83.4		1106	92.79	1116	93.62	
Radiation recode					.010					.046
YES	1549	77.1	1556	80.5		914	76.68	955	80.12	
None/Unknown	459	22.8	376	19.4		278	23.32	237	19.88	
Chemotherapy recode					<.001					.08
YES	1684	83.8	1526	78.9		957	80.29	991	83.14	
No/Unknown	324	16.1	406	21.0		235	19.71	201	16.86	
Primary_site					<.001					<.001
Upper	31	1.5	186	9.6		25	2.10	69	5.79	
Middle	198	9.8	417	21.5		171	14.35	199	16.69	
Lower	1583	78.8	967	50.0		875	73.41	695	58.31	
Other	196	9.7	362	18.7		121	10.15	229	19.21	
Histologic					<.001					<.001
Adenocarcinoma	1381	68.7	777	40.2		682	57.21	603	50.59	
Squamous cell carcinoma	371	18.4	947	49.0		305	25.59	480	40.27	
Other	256	12.7	208	10.7		205	17.20	109	9.14	

The optimal cutoffs for age and tumor size were determined according to X-tile v3.6.1. Most variables did not show remarkably statistical differences between surgery and nonsurgery (*P* > .05) in the postPSM cohort.

PSM = propensity score matching.

**Table 2:** Characteristics of patients before and after PSM according to whether or not PORT.

Characteristics	Entire patients					Propensity-matched patients				
	PORT(+)		PORT(−)		*P* value	PORT (+)		PORT(−)		*P* value
	(n = 322)	%	(n = 1686)	%		(n = 318)	%	(n = 318)	%	
Age					.236					.231
23–66	219	68.0	1085	64.35		216	67.92	212	66.67	
67–74	64	19.88	409	24.26		64	20.13	78	24.53	
75–97	39	12.11	192	11.39		38	11.95	28	8.81	
Gender					.311					.651
Female	43	13.35	265	15.72		43	13.52	48	15.09	
Male	279	86.65	1421	84.28		275	86.48	270	84.91	
Tumor size (mm)					.858					.688
1–44	153	47.52	773	45.85		152	47.80	142	44.65	
45–70	119	37.0	645	38.26		116	36.48	120	37.74	
71–550	50	15.53	268	15.90		50	15.72	56	17.61	
Marital status					.964					.796
Unmarried	101	31.37	531	31.49		98	30.82	94	29.56	
Married	221	68.63	1155	68.51		220	69.18	224	70.44	
Race					.064					.132
White	283	87.89	1523	90.33		280	88.05	277	87.11	
Black	27	8.39	87	5.16		26	8.18	19	5.97	
Other	12	3.73	76	4.51		12	3.77	22	6.91	
Grade					.131					.169
Grade I	16	4.97	66	3.9		16	5.03	6	1.89	
Grade II	117	36.34	662	39.26		115	36.16	117	36.79	
Grade III	178	55.28	930	55.16		176	55.35	186	58.49	
Grade IV	11	3.42	28	1.66		11	3.46	9	2.83	
T stage					.112					.826
T3	268	83.23	1462	86.71		268	84.28	271	85.22	
T4	54	16.77	224	13.29		50	15.72	47	14.78	
N stage					.132					.317
N0	19	5.90	67	3.97		16	5.03	10	3.14	
N1	303	94.10	1619	96.03		302	94.97	308	96.86	
Chemotherapy recode					.005					.796
Yes	287	89.13	1397	82.86		283	88.99	286	89.94	
No	35	10.87	289	17.1		35	11.01	32	10.06	
Primary_site					.06					.190
Upper	8	2.48	23	1.36		8	2.52	3	0.94	
Middle	28	8.70	170	10.08		28	8.81	36	11.32	
Lower	244	75.78	1339	79.4		241	75.79	248	77.99	
Other	42	13.04	154	9.13		41	12.89	31	9.75	
Histologic					.215					.726
Adenocarcinoma	209	64.91	1172	69.51		207	65.09	199	62.58	
Squamous cell carcinoma	64	19.88	307	18.2		63	19.81	64	20.13	
Other	49	15.22	207	12.28		48	15.09	55	17.30	

Although most variables did not show statistical differences between PORT(+) and PORT(−) (*P* > .05), we still performed PSM to remove potential effects of other variables.

PORT = postoperative radiotherapy, PSM = propensity score matching.

**Table 3:** Characteristics of different N stages.

Characteristics	N0		N1		
	(n = 406)	%	（n = 3534）	%	*P* value
Age					<.001
23–66	219	53.94	2029	57.41	
67–74	75	18.47	879	24.87	
75–97	112	27.59	626	17.71	
Tumor size（mm）					<.001
1–44	124	30.54	1449	41.00	
45–70	176	43.35	1395	39.47	
71–550	106	26.11	690	19.52	
Chemotherapy					<.001
No	150	36.95	580	16.41	
Yes	256	63.05	2954	83.59	
Radiation					<.001
No	137	33.74	698	19.75	
Yes	269	66.26	2836	80.25	
Surgery					<.001
No	320	78.82	1612	45.61	
Yes	86	21.18	1922	54.39	
PORT					<.001
Nonsurgery	320	78.82	1612	45.61	
Yes	67	16.50	303	8.57	
No	19	4.68	1619	45.81	
Grade					.001
I	30	7.39	168	4.75	
II	184	45.32	1438	40.69	
III	179	44.09	1865	52.77	
IV	13	3.20	63	1.78	
Sex					<.001
Female	115	28.33	649	18.36	
Male	291	71.67	2885	81.64	
Race					<.001
White	298	73.40	3014	85.29	
Black	81	19.95	316	8.94	
Other	27	6.65	204	5.77	
Histologic					<.001
Adenocarcinoma	139	34.24	2019	57.13	
Squamous cell carcinoma	218	53.69	1100	31.13	
Other	49	12.07	415	11.74	
Primary site					<.001
Upper	49	12.07	168	4.75	
Middle	89	21.92	526	14.88	
Lower	184	45.32	2366	66.95	
Other	84	20.69	474	13.41	
Marital status					<.001
Unmarried	207	50.99	1286	36.39	
Married	199	49.01	2248	63.61	

All variables show statistical differences between N0 stage and N1 stage.

PORT = postoperative radiotherapy.

### 3.2. The role of surgery and PORT

**Figure 2.**
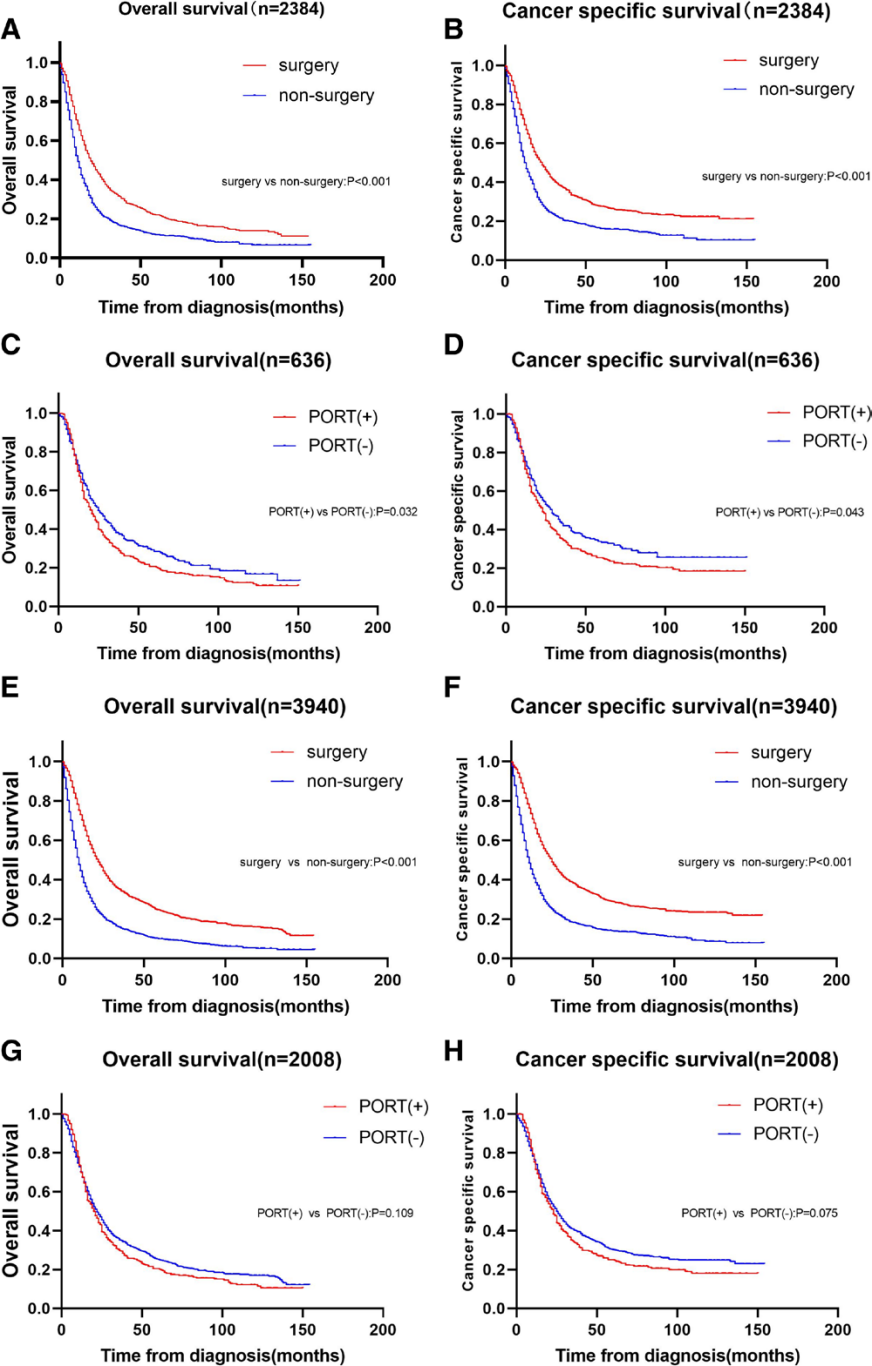
Kaplan–Meier curves before and after PSM in line with whether the surgery is conducted and whether the PORT is carried out. This figure contains (a) overall survival rate (OS) and (b) cancer specific survival rate (CSS) in postPSM cohort in line with whether the surgery is carried out; (c) OS and (d) CSS in postPSM cohort in accordance with whether the PORT is carried out; (e) OS and (f) CSS in pre-PSM cohort according to whether the surgery is carried out; (g) OS and (h) CSS in pre-PSM cohort according to whether the PORT is conducted. PORT = postoperative radiotherapy, PSM = propensity score matching.

The median follow-up in our study was 14 months (range 1–155). During the follow-up period, 3158 cases (80.1%) died, including 2743 cases (69.6%) of esophageal cancer deaths. In the whole cohort, half of patients (n = 2008,51.0%) received surgery, and 16.0% (n = 322)of those undergoing surgery received PORT. In an effort to minimize the influence of other variables, we conducted 1:1 PSM analysis according to surgery or nonsurgery and whether the PORT is conducted. Tables 1 and 2 illustrates the balance of variables before and after PSM in accordance with whether the surgery is conducted and whether the PORT is performed. 1192 patients who underwent surgery were matched with 1192 patients who underwent nonsurgery, and 316 patients who underwent PORT were matched with 316 patients. There were no striking differences in clinical characteristics were observed for most variables in the matched population. In the postPSM population in line with whether the surgery is carried out, the median OS of surgery and nonsurgery were respectively 19.0 (95% CI: 17.2–20.8) and 11.0 (95% CI: 10.2–11.8) months, and the median CSS were respectively 23.0 (95% CI: 20.7–25.3) and 12.0 (95% CI: 10.9–13.0) months. We observed that OS and CSS were evidently better in surgical patients than in nonsurgical patients (Fig. 2a and b). In the postPSM population in accordance with whether the PORT is conducted, patients who received PORT had strikingly lower median OS and CSS than those who did not (OS 20.0, 95% CI:16.5–23.5 vs 25.0, 95% CI:20.4–29.6, *P* = .032; CSS 23.0, 95% CI:19.8–26.2 vs 29.0, 95% CI:23.4–34.6, *P* = .043) (Fig. 2c and d).We also probed into pre-PSM population in line with surgery or nonsurgery and whether the PORT is carried out, found that The median OS and CSS were conspicuously better in patients undergoing surgery than those did not (OS 22.0, 95% CI:20.6–23.4 vs 10.0, 95% CI:9.4–10.6, *P* < .001; CSS 25.0, 95% CI: 23.2–26.8 vs 11.0, 95% CI: 10.3–11.7, *P* < .001) (Fig. 2e and f) and the median OS and CSS were lower in patients undergoing PORT than those did not (OS 20.0, 95% CI:16.7–23.3 vs 22.0, 95% CI:20.4–23.6,*P* = .109; CSS 23.0, 95% CI: 20.0–26.0 vs 25.0, 95% CI: 23.0–27.0, *P* = .075) (Fig. 2g and h). Hence, our results demonstrated that surgery can augment patients’ survival rate, whereas PORT did not heighten patients’ survival rate and even decreased OS and CSS.

### 3.3. Stratified analysis on OS and CSS

**Figure 3.**
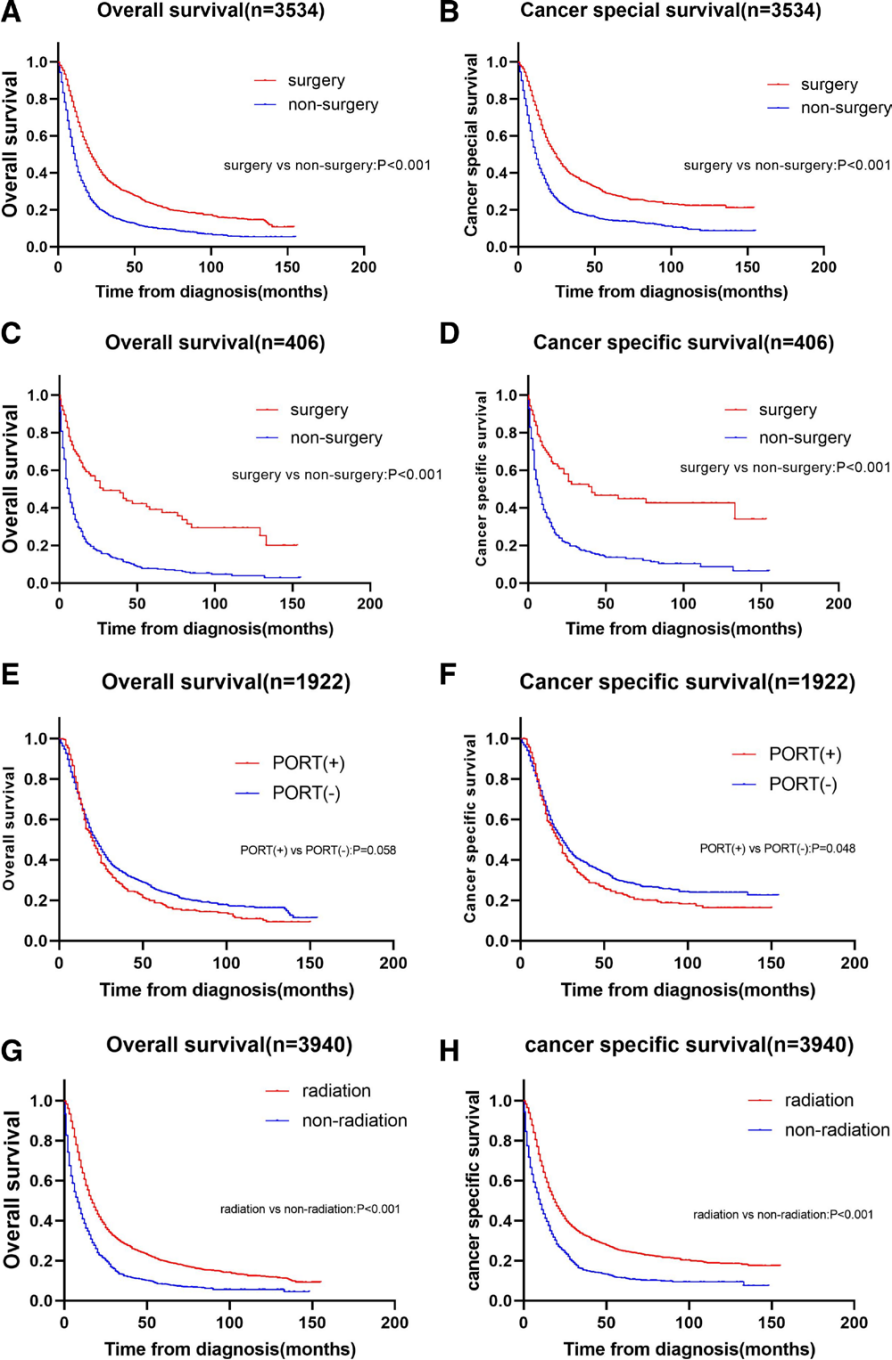
Kaplan–Meier curves for stratified analysis. This figure contains (a) overall survival rate (OS) and (b) cancer specific survival rate (CSS) in esophageal cancer patients with and without surgery in stage N1; (c) OS and (d) CSS in esophageal cancer patients with and without surgery in stage N0; (e) OS and (f) CSS in esophageal cancer patients with and without PORT in stage N1; (g) OS and (h) CSS in all 3940 esophageal cancer patients with and without radiation. PORT = postoperative radiotherapy.

In stratified analysis on the basis of N stage, surgery exhibited OS and CSS benefit for N1 stage (OS HR = 0.548, 95% CI: 0.508–0.590, *P* < .001; CSS HR = 0.534, 95% CI: 0.493–0.579, *P* < .001) (Fig. 3a and b) and N0 stage (OS HR = 0.376, 95% CI: 0.282–0.502, *P* < .001; CSS HR = 0.377, 95% CI: 0.274–0.518, *P* < .001) (Fig. 3c and d). For patients with N1 stage, although the Kaplan–Meier curve demonstrated that the CSS of the nonPORT group was longer than that of the PORT group (*P* = .048), there was no statistically significant difference in CSS between the PORT and nonPORT groups (HR = 1.156, 95% CI: 0.999–1.338, *P* = .051) (Fig. 3f). There are not statistically significant in OS between PORT and nonPORT groups (*P* = .058) (Fig. 3e). In stratified analysis in accordance with whether or not they received radiotherapy, radiotherapy illustrated OS and CSS benefit in patients (OS HR = 0.559, 95% CI: 0.515–0607, *P* < .001; CSS HR = 0.554, 95% CI:0.508–0.605, *P* < .001) (Fig. 3g and h).

### 3.4. Univariate and multivariate analysis

Univariate and multivariate analyses were performed on the postPSM cohort in line with whether the surgery is performed. Univariate analysis manifested that OS and CSS were markedly correlated with age, tumor size, chemotherapy, radiation, surgery, grade and marital status, gender, Primary site and T stage (Table 4). The multivariate analysis on OS revealed that age, tumor size, chemotherapy, radiation, surgery, T stage, grade, gender, marital status independently affected OS (Table 4). The multivariate analysis on CSS revealed that age, tumor size, chemotherapy, radiation, surgery, T stage, grade, gender, marital status and primary site independently affected CSS (Table 4).

**Table 4:** Univariate and multivariate analyses on the postPSM cohort according to whether or not surgery.

Characteristics		OS								CSS							
Univariate analysis	95%CI lower	95%CI upper	*P*	Multivariate analysis	95%CI lower	95%CI upper	*P*	Univariate analysis	95%CI lower	95%CI upper	*P*	Multivariate analysis	95%CI lower	95%CI upper	*P*
Age	23–66	1				1				1				1			
	67–74	1.071	0.963	1.192	.207	1.122	1.005	1.252	.040	1.015	0.904	1.139	.806	1.041	0.925	1.172	.501
	75–97	1.565	1.391	1.761	<.001	1.406	1.242	1.591	<.001	1.475	1.298	1.676	<.001	1.295	1.134	1.480	<.001
Tumor size（mm）	1–44	1				1				1				1			
	45–70	1.023	0.924	1.132	.661	1.027	0.928	1.138	.604	1.095	0.981	1.222	.105	1.094	0.979	1.222	.111
	71–550	1.266	1.123	1.428	<.001	1.354	1.198	1.531	<.001	1.361	1.196	1.549	<.001	1.431	1.255	1.633	<.001
Chemotherapy	No	1				1				1				1			
	Yes	0.441	0.395	0.492	<.001	0.573	0.498	0.660	<.001	0.441	0.392	0.497	<.001	0.557	0.479	0.648	<.001
Radiation	NO	1				1				1				1			
	Yes	0.497	0.448	0.552	<.001	0.631	0.553	0.72	<.001	0.499	0.446	0.558	<.001	0.638	0.553	0.735	<.001
Surgery	NO	1				1				1				1			
	Yes	0.638	0.583	0.699	<.001	0.585	0.533	0.72	<.001	0.615	0.558	0.678	<.001	0.562	0.509	0.622	<.001
N stage	N0	1								1							
	N1	1.035	0.865	1.238	.708					1.024	0.843	1.243	.843				
T stage	T3	1				1				1				1			
	T4	1.123	1.009	1.249	.034	1.140	1.022	1.272	.019	1.159	1.034	1.300	.011	1.172	1.043	1.317	.008
Grade	I	1				1				1			<.001	1			
	II	1.228	0.992	1.52	.060	1.296	1.045	1.607	.018	1.438	1.122	1.843	.004	1.512	1.178	1.941	.001
	III	1.448	1.173	1.787	.001	1.523	1.232	1.884	<.001	1.735	1.358	2.217	<.001	1.809	1.413	2.315	<.001
	IV	1.693	1.184	2.421	.004	1.634	1.141	2.342	.007	2.125	1.439	3.137	<.001	2.035	1.376	3.001	<.001
Sex	Female	1				1				1				1			
	Male	1.215	1.080	1.366	.001	1.347	1.191	1.522	<.001	1.228	1.081	1.395	.002	1.335	1.169	1.525	<.001
Race	White	1				1				1							
	Black	1.228	1.051	1.435	.010	1.217	1.034	1.431	.1018	1.223	1.034	1.447	.019				
	Other	0.995	0.825	1.201	.962	0.982	0.812	1.1186	.848	1.068	0.878	1.301	.510				
Histologic	Adenocarcinoma	1								1							
	Squamous cell carcinoma	0.990	0.896	1.093	.843					0.970	0.871	1.080	.576				
	Other	0.929	0.809	1.066	.294					0.944	0.814	1.094	.445				
Primary site	Upper	1				1				1				1			
	Middle	1.254	0.967	1.626	.087	1.346	1.036	1.749	.026	1.347	1.1012	1.792	.041	1.492	1.119	1.990	.006
	Lower	1.083	0.851	1.377	.517	1.157	0.906	1.477	.243	1.152	0.883	1.504	.297	1.243	0.949	1.628	.114
	Other	1.213	0.934	1.574	.148	1.192	0.916	1.551	.191	1.279	0.959	1.706	.094	1.283	0.960	1.714	.092
Marital status	Unmarried	1				1				1				1			
	Married	0.847	0.733	0.929	<.001	0.812	0.739	0.893	<.001	0.881	0.798	0.973	.012	0.839	0.758	0.929	.001

According to the results of univariate analysis, we included factors with *P* < .05 into multivariate analysis. We found that the independent factors including age, tumor size, chemotherapy, radiation, surgery, T stage, grade, gender and marital status.

CI = confidence interval, PSM = propensity score matching.

### 3.5. Nomogram construction

**Figure 4.**
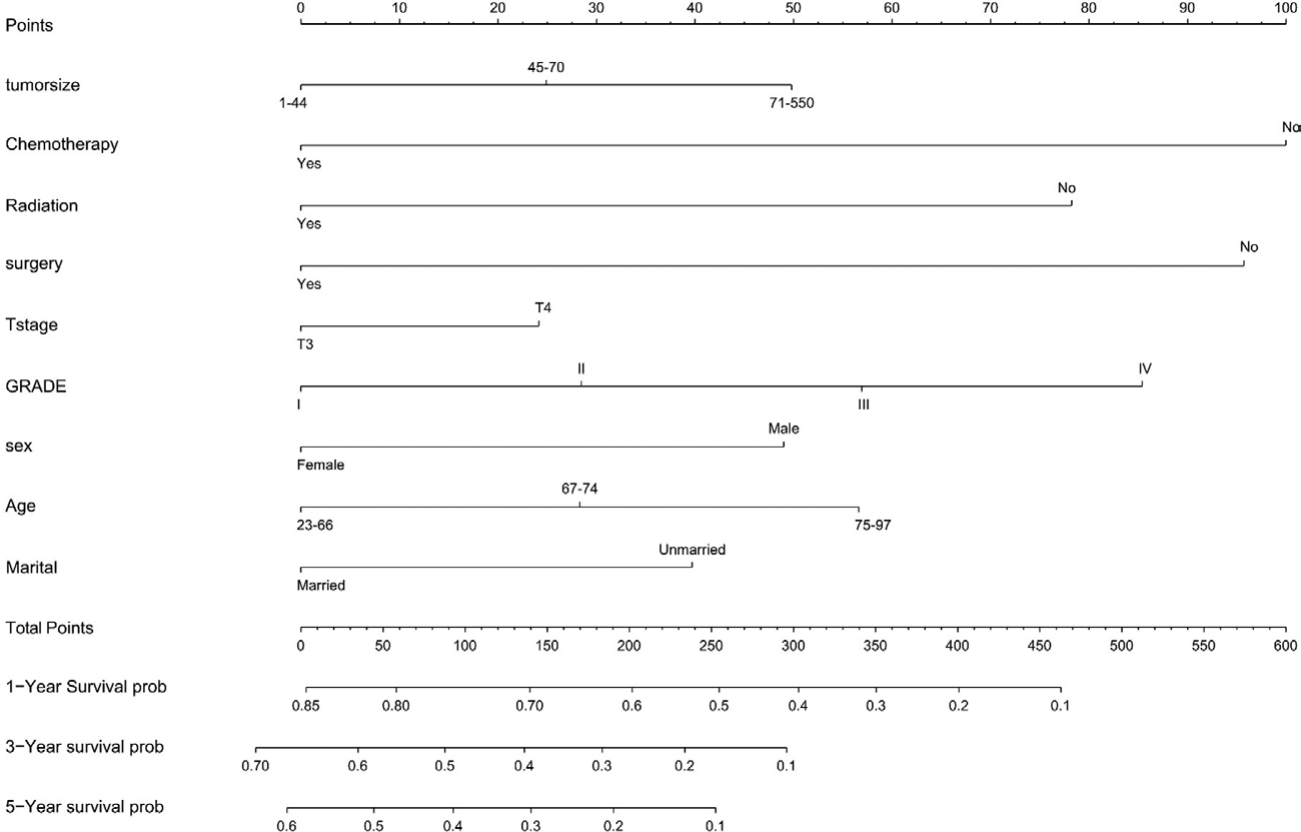
Nomogram for predicting OS in patients with stage III esophageal cancer. We developed a nomogram to predict 5-year survival rate in stage III esophageal cancer patients. The OS nomogram had c-index values of 0.663, indicating low discriminative ability. OS = overall survival rate.

The nomogram was constructed in accordance with the independent factors including age, tumor size, chemotherapy, radiation, surgery, T stage, grade, gender and marital status (Fig. 4). In the nomogram, chemotherapy exerted the most conspicuous influence on prognosis, followed by surgery and grade.

## 4. Discussion

When investigating the data from the SEER database for stage III esophageal cancer diagnosed between 2004 and 2015, we found that surgery noticeably prolonged patients’ survival rate time. Similarly, we also arrived at the same conclusion in N0 and N1 patients. Meanwhile, we found that radiotherapy can better the OS and CSS of patients, but PORT will not ameliorate patients’ survival rate and even lower OS as well as CSS. The value of PORT in patients with esophageal cancer remains controversial. In several studies of patients with PORT, reporting an improvement in the 5-year survival rate in stage III esophageal cancer,^[10]^ noticeably heightened disease-free survival rate in patients with thoracic esophageal squamous cell carcinoma (*P* = .030),^[13]^ an improvement in the 5-year OS (*P* < .001)^[14]^ and an improvement in OS in lymph node-positive patients (*P* < .001).^[15]^ Nonetheless, relevant studies also suggested that PORT did not confer any survival rate benefit compared to nonPORT. As conspicuously revealed by a prospective study, compared with the control group, PORT had shorter overall median survival rate.^[11]^ In another prospective randomized controlled study, there was no statistically significant difference in survival rate between the PORT group and surgery alone.^[16]^ Just as conspicuously illustrated by a meta-analysis on 13 retrospective studies and 6 randomized controlled trials, PORT can ameliorate the OS only in retrospective studies.^[17]^ Nevertheless, most of the aforementioned studies did not stratify patients by stage or had insufficient data or did not take into account the influence of other factors. As a consequence, there may be errors in the detection of the effect of PORT on stage III esophageal cancer. As a result, we conducted this PSM study and found that PORT did not ameliorate patients’ survival rate and even decreased OS and CSS. We matched 318 patients who underwent surgery alone with 318 patients who underwent PORT in an attempt to eliminate the influence of other factors. Apart from that, we obtained similar results when we investigated patients with stage N1 in accordance with whether PORT or not. We did not investigate it due to the deficiency of enough data on patients with stage N0. To investigate whether radiotherapy was responsible for this unusual outcome or PORT, we also explored the effect of radiotherapy on prognosis. Surprisingly, we found that radiotherapy lengthened patients’ survival rate time, while PORT did not elevate or even decreased patients’ survival rate.

One of the most common method for judging the prognosis of cancer is the nomogram.^[18]^ In this retrospective study, we constructed an OS nomogram integrating available information such as age, tumor size, chemotherapy, radiation, surgery, T stage, grade, gender and marital status to predict OS. Previous scholars have constructed a variety of nomogram models for the prognosis of esophageal cancer.^[19–21]^ Nonetheless, a nomogram for stage III esophageal cancer has not yet been constructed, which was the reason why we constructed this nomogram model. The Concordance Index, also known as the c-index, is adopted to evaluate the accuracy of the nomogram model. The c-index >0.7 is considered to have satisfactory predictive ability of nomogram model.^[22]^ The c-index values of this OS nomogram was 0.663, indicating low discriminative ability. Hence, we did not validate the nomogram model. And the model can be employed as a reference tool for decision-making.

It’s essential to discuss several limitations existing in this study. First and foremost, the treatment information in the SEER database is incomplete. The deficiency of crucial information such as radiation dose, chemotherapy dose, patient performance status, and radiation field hindered a more in-depth analysis on the prognostic value of PORT for stage III esophageal cancer. Likewise, although our study revealed that age, tumor size, surgery, T stage, grade, gender and marital status are independent risk factors, the shortage of relevant specific information made further analysis more difficult. Aside from that, the SEER database covers no more than a subset of the entire US patient population, which may not be representative of the entire population.

## 5. Conclusion

As evidently exhibited by the retrospective analysis, surgery can heighten OS and CSS, whereas PORT did not. Nonetheless, given the various limitations of this analysis, caution must be exercised before these findings are universally employed in clinical practice until more randomized trials confirm these results.

## Acknowledgments

The authors thank the participants and their families in this investigation.

## Author contributions

**Conceptualization:** Wenwen Yang, Yanjiang Yang.

**Data curation:** Wenwen Yang, Xiang Ma, Minjie Ma, Biao Han.

**Formal analysis:** Wenwen Yang, Minjie Ma.

**Funding acquisition:** Minjie Ma.

**Investigation:** Wenwen Yang, Yanjiang Yang, Minjie Ma.

**Methodology:** Wenwen Yang, Yanjiang Yang.

**Project administration:** Wenwen Yang.

**Resources:** Wenwen Yang, Yanjiang Yang, Biao Han.

**Software:** Wenwen Yang, Biao Han.

**Supervision:** Wenwen Yang, Biao Han.

**Validation:** Wenwen Yang, Biao Han.

**Visualization:** Wenwen Yang.

**Writing – original draft:** Wenwen Yang.

**Writing – review & editing:** Wenwen Yang, Xiang Ma.
